# A Millian Case for Censoring Vaccine Misinformation

**DOI:** 10.1007/s11673-022-10226-3

**Published:** 2023-01-11

**Authors:** Ben Saunders

**Affiliations:** University of Southampton, Murray Building (#58), Highfield Campus, Southampton, SO17 1BJ United Kingdom

**Keywords:** Censorship, COVID-19, Free speech, J.S. Mill, Misinformation, Vaccines

## Abstract

The spread of vaccine misinformation may contribute to vaccine refusal/hesitancy and consequent harms. Nonetheless, censorship is often rejected on the grounds of free expression. This article examines John Stuart Mill’s influential defence of free expression but finds that his arguments for freedom apply only to normal, reasonably favourable circumstances. In other cases, it may be permissible to restrict freedom, including freedom of speech. Thus, while Mill would ordinarily defend the right to express false views, such as that vaccines cause autism, he might have accepted restrictions on anti-vaccine misinformation during the present pandemic. This illustrates that even the staunchest defenders of free speech can permit temporary restrictions in exceptional circumstances.

Even before the present coronavirus pandemic, there was widespread concern about falling vaccination rates (Brown 2014; Bester 2015; Attwell et al. 2017; Navin and Largent 2017). These concerns are even more pressing since the emergence of the novel coronavirus responsible for COVID-19. At the time of writing (June 2021), many—though of course not all—countries have well-advanced vaccine programmes, but as the numbers vaccinated increase, attention shifts from prioritization in conditions of scarcity (Persad et al. 2020; Giubilini et al. 2021) to ensuring sufficient levels of vaccination. Some have advocated mandatory vaccination or other measures, such as financial incentives (Savulescu 2021) or passports (Wilf-Miron et al. 2021). This paper does not directly engage these proposals. Rather, I am concerned here with how we might combat the spread of vaccine misinformation that contributes towards vaccine hesitancy and refusal in the first place (Kata 2012).

Censorship of misinformation has also been much debated (Martin 2015; Kennedy and Leask 2020; Larson 2020; Armitage 2021; Mills and Sivelä 2021). My contribution in this paper is to argue that even those who ordinarily favour extensive rights of free speech may be prepared to accept restrictions on this in the context of a global pandemic. I illustrate this claim with the example of the nineteenth-century English philosopher, economist, and political reformer John Stuart Mill.

Mill is still well known for his influential defence of individual liberty. His arguments for freedom of discussion are particularly relevant here since they explicitly include the right to air false views (Mill 1977 [1859], 243–252), implying that authorities have no right to suppress opinions—such as the notorious claim that vaccines cause autism—despite their being unfounded or even discredited. This Millian argument is of obvious appeal to those who spread misinformation about vaccines since it allows them to defend their right to do so without having to demonstrate the truth or even plausibility of their claims (Kata 2012, 3783).

However, I argue here that Mill’s famous defence of free discussion does not actually preclude restrictions on such speech *in current pandemic conditions*. He allows that it may be appropriate to restrict when and where particular views are expressed. His most famous example of this concerns the “opinion that corn-dealers are starvers of the poor,” which Mill says might be circulated in print but ought not to be “delivered orally to an excited mob assembled before the house of a corn-dealer” (Mill 1977 [1859], 260). This case illustrates that the freedom of discussion he defends, though extensive, does not apply regardless of context (cf. Jacobson 2000, 287). In fact, his arguments for liberty in general apply only to certain, reasonably favourable, circumstances (Mabsout 2021).

In cases of emergency, such as a global pandemic, it may be permissible to suspend ordinary liberties. This could extend not only to cases such as freedom of movement and association, but perhaps also to freedom of expression. Thus, even if we should ordinarily tolerate vaccine misinformation, this policy need not apply to our present context. In the midst of a pandemic, there may be an exceptional justification for restricting vaccine misinformation. This might permit the imposition of restrictions by governments or other organizations, such as social media platforms—as, for instance, Twitter’s recent decision to suspend U.S. Congresswoman Marjorie Taylor Greene’s personal account (BBC 2022). I will refer to such restrictions as censorship for, while they might only amount to “no-platforming” or restrictions on the context of expression, I take it that they go beyond what would ordinarily be justified.

This argument is limited in scope since it only shows a right to censor misinformation during an emergency and not in more normal times. Nonetheless, it is significant because it establishes that *even* Mill might accept some limits on freedom of expression in our present circumstances. Of course, we might reject Mill’s arguments for freedom of discussion, in which case it is easier to justify restrictions on false or misleading expression (Emerick 2021, 135). Nothing that I say here is intended to preclude this possibility. However, my argument is primarily directed towards those who are wary of any interference with expression for broadly Millian reasons. The argument is intended to show that the reasons Mill offers, even if good in more normal circumstances, do not necessarily apply to all circumstances. Thus, acceptance of these arguments does not commit us to tolerating vaccine misinformation during the pandemic even if we would otherwise.

My argument is noteworthy precisely because it starts by granting a strong presumption in favour of free expression, which is a concession towards the purveyors of misinformation. If certain restrictions can still be justified, even on these assumptions favourable to free speech, then the case for them will be all the clearer should we adopt a starting point that is less hospitable towards misinformation. In this respect, my argumentative strategy is like that of Brennan (2018). Brennan argues that the case for vaccine mandates is so strong that even libertarians, who are generally opposed to government interference, ought to accept it. Similarly, my argument is that even those generally hostile to censorship of misinformation—like Mill—might accept restrictions in this special case. If this is so, then the case for these restrictions must be compelling.

The argument is also limited in another way, in that it only addresses whether or not interference is morally legitimate. I do not discuss how feasible or efficacious restrictions may be. These are real concerns. Given that much misinformation circulates through social media sites (Kata 2012), it may be much harder to regulate than print or broadcast media. The arguments canvassed here concerning the legitimacy of censorship apply to all forms of expression, although pragmatic considerations may differ from case to case. Further, there is a reasonable worry that attempts to censor or suppress certain information may diminish trust in governments (Larson 2020). It could be that some measures are counterproductive, in which case they are of course ill-advised (Bester 2015). Finally, there are important questions over whether governments (or other organizations) can be trusted with the power of censorship, especially given that some governments have themselves been accused of spreading messages that are confusing, misleading, or even false (Newton 2020; Shaw 2021).

What measures are possible or effective is likely to vary from context to context. These questions are beyond the scope of the present paper. However, such practical questions would be immaterial if it were always wrong to restrict speech. Thus, my focus is on whether the government (or some other authority) has a *right* to restrict speech, not with whether it is expedient to exercise that right. It might turn out that it is better as a matter of policy not to censor misinformation. That is a question for another time. My claim here is only that it would not be wrong in principle.

## The Relevance of John Stuart Mill

John Stuart Mill’s 1859 essay *On Liberty* has been described as “perhaps the most eloquent defense of individual liberty ever written” (Riley 1990, 27). It argues that the only justification for restricting a competent adult’s freedom is to prevent harm to others, thereby ruling out (inter alia) paternalistic interference. While not everyone accepts this “harm principle,” it is still widely invoked in debates over state interference and continues to be applied to contemporary issues such as smoking bans (Silva 2011), alcohol pricing (Saunders 2013), religious education (du Plessis 2016), mental health acts (Browne 2016), and pandemic responses (F.G. Miller 2021).

Of particular relevance here is Mill’s chapter two, “Of the liberty of thought and discussion,” which itself “has become an indispensable part of Western intellectual tradition” (Peonidis 2002, 606). The connection between this discussion and the rest of the work is controversial; a number of interpreters have argued that this chapter is something of a digression, since Mill’s arguments for free speech seem to be independent of his harm principle (Day 2000; Riley 2005), though Dale Miller (D.E. Miller 2021) has recently defended the continuity of Mill’s argument. Whatever the answer to this exegetical conundrum, Mill remains influential in discussions of free expression, including Holocaust denial (McKinnon 2007; Schauer 2012), hate speech (Brink 2001; Brown 2008), and pornography (Vernon 1996; Cowen 2016).

Mill is often cited “as an advocate for unrestricted freedom of discussion” (Turner 2021, 125). I argue below that it is not entirely accurate (though I am not the first to make such an argument). However, I only argue that he *might* have accepted restrictions on vaccine disinformation, not that he would actually have done so. I do not claim that this is the only, or even the best, interpretation of everything that Mill had to say on the subject of free speech. He is notorious for expressing his ideas differently in different places (Jacobson 2000). Given the complexity and nuance of Mill’s thought, it is possible for selective readings to support opposing positions (Mabsout 2021). Establishing which position best reflects Mill’s considered views is difficult. Nonetheless, I show that contextual restrictions are consistent with at least *one prominent strand* of Mill’s thought.

In this respect, the interpretive claim that I am making is similar in kind to that recently advanced by J.P. Messina, who also focuses on one particular aspect of Mill’s thought—what Messina (2020, 5) calls Mill’s “darker side”—without claiming that this coheres with everything that Mill says on the subject or denying that there are other sides to Mill’s thought. Likewise, I do not claim to give a complete and balanced picture of Mill’s views but only to emphasize elements of his thought that might lead in this direction. Thus, the case for censorship that I offer is *Millian*, in the sense that it is derived from Mill’s thought but not necessarily one that Mill himself would endorse.

## Mill’s Arguments for Freedom of Expression

Mill’s argument against censorship of discussion comes in three parts. First, it is possible that the opinion to be suppressed is true. Mill points out that humans, including those who wish to censor opposing views, are fallible and may be mistaken no matter how certain they feel. While we are entitled to act on our own beliefs, we have no right to decide matters for other people. Rather, we should allow the contestation of our beliefs so that our errors can be corrected. The best warrant that we have for any of our beliefs is “a standing invitation to the whole world to prove them unfounded” (Mill 1977 [1859], 232).

Second, Mill argues that censorship is usually unjustified *even if the opinion to be censored is entirely false* (Mill 1977[1859], 242). Even if we somehow, impossibly, *knew* that we were right with absolute certainty, and not merely our own feeling of certainty, it would still be unjustified to silence rival views. If we do this, then we will no longer need to defend and justify our beliefs, so they risk becoming mere prejudice or superstition (Mill 1977 [1859], 244), learned by rote but not truly understood (Mill 1977 [1859], 247). Mill argues that it is important for people not only to have true beliefs but to understand the grounds for these beliefs, since “beliefs not grounded on conviction are apt to give way before the slightest semblance of an argument” (Mill 1977 [1859], 244). Thus, even false opinions, though they cannot contribute to the truth of our beliefs, can still contribute to our proper understanding and appreciation of our true beliefs.

Together, these arguments suggest that we have powerful reasons not to censor statements such as “vaccines cause autism.” This alleged link received much publicity due to a study of the MMR vaccine by Andrew Wakefield et al. published in *The Lancet*, although subsequent studies found no evidence of a causal link (Farrington et al. 2001; DeStefano 2007). Ten of the thirteen authors of the original study later retracted this interpretation of the results (Murch et al. 2004) and, indeed, the paper itself was subsequently fully retracted from the published record (Editors of *The Lancet* 2010). Nonetheless, despite its findings being discredited, this study has continued to fuel vaccine hesitancy.

If we accept Mill’s arguments above, then we ought not to censor such statements, whether or not they are true. The fact that many people have looked for a link between vaccines and autism but not found one is evidence that there is not one (Pickering 2015). However, we should remember that we are not infallible. No matter how strong the current evidence against a link, it does not preclude the possibility that new evidence of a link might emerge. Recent interpreters have emphasized the importance that Mill attaches to openness to challenge (Shah 2021; Thomas Wright 2021). No matter how sure we are of our opinion, we must allow the other side to be heard, since it is only by hearing the other side of the argument that we have either “the opportunity of exchanging error for truth” or “what is almost as great a benefit, the clearer perception and livelier impression of truth, produced by its collision with error” (Mill 1977 [1859], 229).

These arguments present a powerful challenge to any proposals for censorship, to which Mill adds a third, intermediate, case. It may be that the opinions to be censored contain “a part of the truth; sometimes a greater, sometimes a smaller part, but exaggerated, distorted, and disjointed from the [other] truths by which they ought to be accompanied and limited” (Mill 1977 [1859], 252). Popular opinions, he suggests, are often part of the truth, but not the whole truth. For instance, someone might believe that they do not need a certain vaccine because they will be protected by herd immunity.

There is indeed *some* truth in this line of thought, at least where vaccines block transmission. How far this is applicable to COVID vaccines is not yet entirely clear. It seems even the double-vaccinated can transmit the virus (Singanayagam et al. 2021), though it may be that transmission is reduced. Nonetheless, for some other diseases, if everyone else were vaccinated against the disease in question, then the individual in question would likely be safe. However, herd immunity should not be taken for granted. If too many people reasoned like this and therefore refused vaccination, then herd immunity would soon be undermined (Giubilini 2020). Thus, this thought is potentially misleading if taken to imply that one does not need vaccination. Where prevailing opinion is similarly one-sided, then “the nonconforming opinion is needed to supply the remainder of the truth, of which the received doctrine embodies only a part” (Mill 1977 [1859], 252).

Though not everyone accepts Mill’s arguments for freedom of expression, they continue to be influential in contemporary discussions. In particular, they are likely to appeal to opponents of censorship, since there is no onus on them to prove that their statements are true or even plausible in order to defend their right to free speech. However, while Mill’s arguments favour allowing misinformation in normal circumstances, they are limited in their scope of application. Though Mill favours freedom of expression as a general policy, he acknowledges that there may be grounds to impose some restrictions in exceptional contexts. Given that the present pandemic is such an exceptional context, we may currently be justified—even in Mill’s eyes—in censoring or restricting vaccine misinformation even if we should normally permit it for the reasons outlined above.

## The Limits of Freedom

Mill’s defence of liberty is not for everyone but applies only “when certain social and cognitive conditions are satisfied” (Mabsout 2021, 1). In particular, it is intended for “human beings in the maturity of their faculties,” excluding minors, otherwise incompetent individuals, which may include the mentally ill (Browne 2016), and—more controversially—those in what Mill calls “backward” societies (Mill 1977 [1859], 224). Until people are capable of listening to reason, it is sometimes necessary and appropriate to exercise benevolent despotism over them for their own good. This qualification suggests a difference in treatment before and after some moment of maturity, such as the legal age of adulthood. Beyond that point, the harm principle precludes paternalistic coercion but, until this point is reached, such coercion is justifiable.

Of course, this is a rather simplistic picture of intellectual or moral maturity. In reality, different individuals mature at different rates. Further, a given individual may be sufficiently competent to make some decisions and not others (Browne 2016). Moreover, competence does not uniformly increase over time. Though Mill does not discuss old-age cognitive decline, we may think that the principle of liberty *ceases* to apply in some cases, where a person is no longer competent to manage their own affairs. It may then be permissible for others to interfere with their self-regarding choices (Browne et al. 2002), much as it would be permissible to interfere with the choices of a child. Again, this point may vary from person to person and decision to decision.

However, Mill *does* acknowledge that anyone may suffer from temporary incompetence when he gives a fuller list of exclusions in a later example. In discussing whether someone should be allowed to cross an unsafe bridge, he suggests that the individual—once informed of the danger—should be left to choose for himself whether to proceed, “unless he is a child, or delirious, or in some state of excitement or absorption incompatible with the full use of the reflecting faculty” (Mill 1977 [1859], 294). Here, then, he recognizes that it is permissible to restrict people’s freedom of action during temporary episodes of incompetence even if they are normally competent adults.

Though these qualifications may not immediately appear relevant to freedom of expression, the same reasons that justify intervention in these cases may also justify restrictions on misinformation. Mill’s case for freedom of expression hinges not only on the interests of speakers in expressing their opinions but also on the interests of their audience in being exposed to different views (Riley 2005). However, the value of this exposure depends on the audience’s capacity to reflect critically on the ideas presented to them. Even ardent advocates of free speech usually recognize that some things ought not to be said to (or even in front of) children, although those things are not objectionable in themselves.

Mill recognizes that some things, not wrong in themselves, may be “offences against decency” when done in public (Mill 1977 [1859], 296). Further, as we have seen, he adds that inflammatory opinions should not be “delivered orally to an excited mob” even if the very same opinions “ought to be unmolested when simply circulated through the press” (Mill 1977 [1859], 260). In the former case, such remarks could easily incite a riot, without those involved having the chance to reflect or deliberate on what was said (D.E. Miller 2021, 138–139). Hence, while expression should not be restricted on grounds of its *content*, even if it is false or immoral, it is sometimes permissible to restrict the *context* of expression.

Thus, Mill’s argument for freedom, both of action and expression, does not apply to all times and places. It is intended only for competent adults in civilized communities (Mill 1977 [1859], 223–224). Where circumstances are less favourable, for instance because people have temporarily lost the capacity to reason, it may be necessary and appropriate to abridge these freedoms. This might mean that an individual’s freedom may be restricted, for instance when they are in a temporary state of excitement. But it might also be appropriate to suspend freedoms more generally in times of crisis or national emergency (Turner 2021). Mill not only held that a benevolent despotism may be necessary in uncivilized societies but also that a “temporary dictatorship” may be necessary in any country “in cases of extreme exigency” (Mill 1977 [1861], 403). In both cases, restrictions on freedom should be tolerated only for as long as necessary to bring about more favourable conditions but, so long as they are required, they are justifiable.

It might be objected that restricting the scope of the harm principle in this way ends up removing any real constraint on interference. Behavioural social science suggests that people are not really the rational and autonomous choosers postulated by economic theory. People’s choices are often the result of psychological biases. Their choice of A over B might simply be the result of the way in which options are presented or described rather than reflecting any deliberative preference. It might thus be argued that these findings open the door to widespread paternalistic interference in our actual world (Conly 2013). However, I am *not* saying that the harm principle only protects the freedom of highly idealized “rational agents” possessing full information, free of bias, and so on. Mill was aware of human psychological limitations and qualified his defence of freedom accordingly (Mabsout 2021). Nonetheless, he clearly intended it to apply to most ordinary adults in typical, non-idealized circumstances.

My point is merely that the harm principle may not apply in especially unfavourable circumstances, where people lack their ordinary capacities for reflective choice, such as the aforementioned “state of excitement or absorption” (Mill 1977 [1859], 294). This does not mean that it ceases to apply whenever circumstances are less than fully optimal. Of course, this raises questions regarding when circumstances are “good enough” for rational deliberation and when they are not. I do not attempt to specify this threshold here, though I assume that “good enough” falls some way short of optimal or ideal. It should be low enough that most adults can generally be presumed to meet it most of the time, unless there is good evidence to conclude otherwise. However, where someone’s capacity for choice is impaired, temporarily or permanently, the harm principle may cease to apply. If they can no longer be presumed the best judge of their own interests, then it may sometimes be justifiable to interfere with their freedom.

## Application

We should doubtless be wary of governments that too readily invoke crisis in order to justify far-reaching “emergency powers.” Nonetheless, the global coronavirus pandemic surely counts as a case of extreme exigency. In such circumstances, it may be necessary to restrict individual freedom in order to protect public health (Pierce 2011). Indeed, many governments responded to the pandemic by imposing some form of “lockdown” measures, restricting freedoms of movement and assembly to various degrees. Though these lockdowns interfere with people’s freedom to carry out what are usually everyday activities, such as work, shopping, and socializing, many of these restrictions are compatible with the harm principle, provided the aim is to prevent harm to others by reducing transmission of the virus (F.G. Miller 2021). These measures are not exceptions to the harm principle but merely serve to show how its implications can vary in different circumstances.

In the context of a pandemic, activities that would not usually cause harm to others can become dangerous and thus potentially liable to restriction. This does not mean that the restrictions we have seen are always justified. There is still room to debate whether or not any particular response is effective or proportionate. For instance, it has been argued that it would be better to impose a targeted lockdown of vulnerable groups, shielding them from harm, rather than restricting everyone (Savulescu and Cameron 2020).

Whether any given interference is justified or not is a further question, depending on issues of effectiveness, necessity, and proportionality. For Mill, the answer ultimately depends on utilitarian cost-benefit analysis, rather than on the harm principle itself. The harm principle tells us that “society has jurisdiction over” “any part of a person’s conduct [that] affects prejudicially the interests of others,” but this means only that “the question whether the general welfare will or will not be promoted by interfering with it, becomes open to discussion” (Mill 1977 [1859], 276). It must still be considered whether “the attempt to exercise control would produce other evils, greater than those which it would prevent” (Mill 1977 [1859], 225). Thus, we might think of the harm principle as only the first part of a two-stage process (Turner 2014; D.E. Miller 2021). My concern here is only with this first stage—that is, with whether state interference is potentially justifiable, and not with whether it is actually justified. The answer to the latter question will depend on the particular context.

I suggest that the ordinary right to express anti-vaccine misinformation could also be suspended during the pandemic. Mill’s arguments for freedom of expression highlight how even the propagation of false views can be beneficial, at least in the long run, but this assumes that there is scope for debate and reflection. Actual public debate may fall short of Mill’s ideal of rational discussion (Peonidis 2002). Again, merely being less than ideal is not sufficient justification for censorship. But, where the circumstances are sufficiently bad for deliberation, certain restrictions may be justified, at least until conditions are more favourable. I take it that this is why inflammatory remarks should not be made in front of an excited mob (Mill 1977 [1859], 260)—because in this context they are likely to lead immediately to harm.

The reasoning here is similar to that employed by U.S. Supreme Court Justice Holmes, who famously concluded that the right to free expression does not extend to falsely shouting “fire” in a crowded theatre (McKinnon 2007). Not only would this predictably cause a stampede, but it would not be easy in the ensuing panic to convey that it was a false alarm (McKinnon 2016). Thus, restrictions on expression are justified in these cases, not because of the content of what is expressed, but by features of the circumstances—that is, because of “the deficits of the situation when viewed as a forum for deliberation” (Niesen 2019, 15).

My contention is that the midst of a global pandemic is, similarly, not an appropriate forum for reasoned debate on the merits of vaccination. So, even if Mill’s arguments provide good reasons for us to permit vaccine misinformation in ordinary circumstances, before or after the pandemic, these reasons may be inapplicable during the context of a pandemic. In this situation, like that of the excited mob, people may not be in a position to reflect calmly and critically on the claims that they hear. Further, allegations leading to increased vaccine refusal—or even hesitancy—could contribute towards further harms, including deaths, in the meantime. Even if most people would eventually choose to vaccinate after hearing both sides of the debate, this discussion would cause significant delay and therefore greater harm at a time when speedy action is needed (cf. McKinnon 2017).

To be sure, there is one potentially important difference between the riot case that Mill discusses and the case of vaccine misinformation. In the former, inflammatory speech may incite people to *do* something (riot) that causes harm. In the latter, it might be argued that vaccine misinformation only causes people *not* to do something. However, Mill holds that people can be held to account for harm caused (or allowed) by inaction as well as that caused by action (Mill 1977 [1859], 225). Thus, the harm principle may license interference with vaccine refusers. More to the point though, we are not talking here of interfering with the vaccine refusers themselves but rather with those who spread misinformation about vaccines. This is an action that, like incitement, may prompt others to do something harmful (refuse or delay vaccination).

It might be objected that speakers should not be held responsible for “indirect” harms that come about via the agency of others. If Robert’s claim about vaccines leads Dahlia to refuse or delay vaccination, and this turns out to be harmful because she ends up transmitting the virus to others, it would ordinarily be Dahlia—not Robert—held responsible for her (in)action. However, this assumes that Dahlia was “able to deliberate and decide for herself” (D.E. Miller 2021, 135). To the extent that this is not true, perhaps Robert *should* bear some responsibility for the consequences of his action, at least if he knew that Dahlia was likely to act on his suggestion. In any case, our concern is not with Robert’s culpability but with whether his action is liable to interference. The case for interfering with his speech seems stronger if Dahlia is not capable of deliberating than it would be if she were (in which case, she—not Robert—would be the cause of subsequent harm). This interference would be in keeping with the proposal that the state can “exclude the influence of solicitations … which the State believes to be wrong … [so] that persons shall make their election, either wisely or foolishly, on their own prompting” (Mill 1977 [1859], 297).

As we have seen, Mill’s defence of freedom assumes reasonably favourable circumstances in which agents can deliberate on claims that they are presented with and make up their own minds. As noted above, these conditions are not intended to be particularly idealistic or demanding, but they do suggest that his arguments for freedom may be inapplicable when conditions are especially unfavourable to deliberation. This is something that Mill recognizes, proposing that speech that would be permissible elsewhere may nonetheless be limited in certain contexts.

Since Mill does not offer a full account of the contexts in which expression may or may not be restricted, it is difficult to know how far he would take this. Emerick (2021) argues that Millian conclusions might apply to utopian societies in which all parties are free and equal but are irrelevant in actual societies, marked as they are by injustice and unequal power relations. Perhaps Mill underestimated the challenges here, though it is worth noting that he was concerned with ensuring a fair hearing for minority viewpoints (e.g., Mill 1977 [1859], 254) and sought to do this through measures such as expansion of the franchise (Mill 1977 [1861], 467ff) and proportional representation (Mill 1977 [1861], 448ff).

Of course, some possible restrictions are clearly excessive, even if they only concern the context of speech and not its content. For instance, if people were prohibited to speak except between the hours of 4 and 5 a.m. on Tuesdays or in their own homes. Both of these examples only involve restrictions on when or where people speak rather than what they say. Nonetheless, I assume that they are unacceptable (both to Mill and in fact). To be clear, I do not mean to suggest that Mill would accept *all* restrictions on context but only to emphasize that he accepted *some* restrictions on context as legitimate. Thus, it is possible that current circumstances are sufficiently exceptional that the ordinary reasons for freedom of discussion do not apply.

While Mill does not offer a full account of the circumstances that may justify restrictions, the examples that he does give suggest that they include cases unfavourable to deliberation and, in particular, emergency situations. A global pandemic, I submit, is an exceptional circumstance in which people may not be able to deliberate rationally about vaccines or—even if they can—where time spent in such debate causes harmful delays when immediate action is needed. Thus, it is possible that Mill would allow interference with vaccine misinformation in this context, even though he would oppose this in normal circumstances. Since this censorship is justified only by a state of emergency, it should only last as long as required and not become a “new normal.” As soon as conditions are sufficiently normal, then Mill’s arguments for freedom of discussion would preclude continued interference. But, until then, exceptional circumstances may justify exceptional measures.

## Conclusion

The spread of misinformation about vaccines is a cause for concern. The ethics of censorship has been much debated, especially of late (Martin 2015; Kennedy and Leask 2020; Larson 2020; Armitage 2021; Mills and Sivelä 2021). Faced with such threats, one option for purveyors of misinformation is to argue that their claims are true or, at least, not proven false (Kata 2012). Another is to appeal to their right—either legal or moral—to free expression (Emerick 2021). This latter argument is more robust, since it would defend the right to speech even if it is false.

Purveyors of misinformation may think that they can turn to Mill’s influential defence of free speech in order to support their right to free expression. However, I have argued that even Mill—and contemporary liberals influenced by his arguments—might accept restrictions on such speech in exceptional circumstances. The arguments of *On Liberty* protect freedom of speech and self-regarding action for competent adults, yet Mill introduces various qualifications and caveats suggesting that these freedoms can be limited in less favourable circumstances. Restrictions may be necessary not only in uncivilized societies but also “in cases of extreme exigency” (Mill 1977 [1861], 403).

I have not, however, argued for any particular restrictions. What measures, if any, might be feasible and effective will depend upon empirical considerations. In some cases, particularly where trust in government is low, it may be that censorship of misinformation would be counterproductive, serving only to strengthen anti-vaxxer conspiracies. Messina (2020) emphasizes Mill’s fear that any state power, including censorship, could be abused. Even in other cases, there is a legitimate question over what justifies a government to silence dissenting views. If we accept that governments may be wrong even when guided by scientific experts, then there is a danger that even well-meaning censors could be suppressing truth. Shah (2021) argues that censorship is self-undermining because, according to Mill, it deprives us of rational justification for our beliefs. However, if these arguments point towards a universal prohibition on censorship, then they prove too much.

Mill evidently thought that censorship *could* be legitimate in certain circumstances. He did not, so far as I am aware, explain how it is that we may trust the censors in those cases. Perhaps he was optimistic that, since these powers are only temporary, the prospect of future accountability would ensure responsibility. Maybe he thought that in a genuine emergency abuse of dictatorial power was the lesser evil. Whatever the explanation, Mill clearly held that the extensive liberty of action and speech that he defended was not suitable for all circumstances and could (indeed, should) sometimes be curtailed. Thus, while dissenting opinions should ordinarily be permitted—and perhaps even promoted or encouraged—this need not apply in special cases where the expression of certain views cannot have its usual salutary effects and may even cause significant harm. In these circumstances, temporary limits on time or place of discussion may be justifiable.

My claim here is that the pandemic may be such a situation, permitting special restrictions on what should ordinarily be tolerated. Misinformation is likely to lead people to refuse or delay vaccination. This misinformation might, in time, be corrected by more speech. This would ordinarily be Mill’s favoured option, if action were not urgently needed. However, he acknowledged that the triumph of truth over falsehood need not be quick (Mill 1977[1859], 238–239). In the present circumstances, delays are likely to lead to significant harms, both in deaths from coronavirus and prolongation of lockdown and other measures (Savulescu 2021). Given these costs of delay, more speech is not able to prevent these harms. In this context, exceptional action may be necessary to prevent harm. Thus, even Mill—the celebrated liberal champion of free speech—might have accepted *temporary* restrictions on vaccine misinformation until the immediate danger has passed and conditions are once again more favourable for discussion.
